# Psychometric properties of responses by clinicians and older adults to a 6-item Hebrew version of the Hamilton Depression Rating Scale (HAM-D_6_)

**DOI:** 10.1186/1471-244X-13-2

**Published:** 2013-01-03

**Authors:** Yaacov G Bachner, Norm O’Rourke, Margalit Goldfracht, Per Bech, Liat Ayalon

**Affiliations:** 1 Department of Public Health and the Center for Multidisciplinary Research in Aging, Faculty of Health Sciences, Ben-Gurion University of the Negev, P.O.B 653, Beer-Sheva 84105, Israel; 2 Faculty of Arts and Social Sciences, Simon Fraser University, Burnaby, (BC), Canada; 3 Community Division, Clalit Health Services, Tel Aviv, Israel and Department of Family, Health Care, Bruce Rappaport Faculty of Medicine, The Technion, Haifa, Israel; 4 Department of Psychiatry, Frederiksborg General Hospital, Hilleord, Denmark; 5 Louis and Gabi Weisfeld School of Social Work, Bar-Ilan University, Ramat Gan, Israel

**Keywords:** Depression, Hamilton depression rating scale, Hebrew, Elderly

## Abstract

**Background:**

The Hamilton Depression Rating Scale (HAM-D) is commonly used as a screening instrument, as a continuous measure of change in depressive symptoms over time, and as a means to compare the relative efficacy of treatments. Among several abridged versions, the 6-item HAM-D_6_ is used most widely in large degree because of its good psychometric properties. The current study compares both self-report and clinician-rated versions of the Hebrew version of this scale_._

**Methods:**

A total of 153 Israelis 75 years of age on average participated in this study. The HAM-D_6_ was examined using confirmatory factor analytic (CFA) models separately for both patient and clinician responses.

**Results:**

Reponses to the HAM-D_6_ suggest that this instrument measures a unidimensional construct with each of the scales’ six items contributing significantly to the measurement. Comparisons between self-report and clinician versions indicate that responses do not significantly differ for 4 of the 6 items. Moreover, 100% sensitivity (and 91% specificity) was found between patient HAM-D_6_ responses and clinician diagnoses of depression.

**Conclusion:**

These results indicate that the Hebrew HAM-D_6_ can be used to measure and screen for depressive symptoms among elderly patients.

## Background

Depression is a common debilitative psychiatric condition ranked high in prevalence among all mental health conditions [1]. Lifetime prevalence may be as high as 20% [2] and, at any one time, 5–10% of the world’s population meets diagnostic criteria for a major depressive episode [3]. Depression is projected to be the second leading cause of disability worldwide in 2020 [4].

Clinical depression is common in primary care with rates of prevalence among older adults ranging between 4–24% [5,6]. Untreated elderly patients are at higher risk of morbidity and mortality [7] and experience slower rates of recovery [6,8]. Moreover, chronic depression is a significant risk factor for dementia [9].

Given that depression is amenable to treatment, valid and reliable screening tools are necessary to identify this patient population. Among existing instruments, the clinician-administered Hamilton Depression Rating Scale (HAM-D) was first developed to assess the efficacy of the first generation of antidepressant medications [10]; the HAM-D has since become the gold standard for measuring symptom severity and change in randomized clinical trials. Among various formats (17, 21, 24 & 28 items) [10,11], the 17-item (HAM-D_17_) has been used most frequently. Scale items measure mood, insomnia, anhedonia, agitation, gastro-intestinal and other somatic symptoms, weight change, suicidal ideation, hypochondriasis, anosognosia, and psychomotor and cognitive retardation.

Despite widespread usage, various researchers have questioned whether the HAM-D_17_ is a unidimensional or multidimensional instrument [12-15]. This is problematic as multi-factorial measurement may impede the detection of symptom change over time, treatment response characteristics [16] and the ability to distinguish the relative efficacy of treatments [13]. This assertion is supported by meta-analytic study findings indicating that certain scale items are less sensitive to measurement of symptom severity. In addition, some items have comparatively poor inter-rater and retest reliability, and the response-option format may not be optimal [17]. In light of these findings, some have suggested that the 17-item HAM-D may be less than ideal for clinical research applications [14,15,17,18].

These limitations have led researchers to propose abridged versions of the HAM-D that are quick to administer yet sensitive to measurement of symptom levels, change over time and relative differences in treatment efficacy. For instance, Maier and Philipp [19] proposed a 6-item version of the HAM-D. More recently, an 8-item version was devised by Gibbons and colleagues [20] by applying item response theory. Research to date suggests that both versions are sensitive to change over time and can identify patients in remission [21,22]. Recently, a scale consisting of 7 items was also suggested [23]. The items were empirically identified on the basis of response frequency and sensitivity to change of the individual HAM-D items with depressed samples [24].

Among the abridged versions of the Hamilton scale, the most frequently used was developed by Bech et al. (HAM-D_6_) [25]. Using item analysis, these researchers [25] have proposed a 6-item HAM-D as a unidimensional measure of depressive symptomatology [14]. This HAM-D_6_ is composed of items measuring core symptoms of depression (i.e., depressed mood, self-esteem and feelings of guilt, social interaction and interests, psychomotor retardation, anxiety, and somatic symptoms). Compared to the HAM-D_17_, this assessment appears to measure a unidimensional construct [13-15,17,25,26], and it is as sensitive [14] or more sensitive in detecting drug–placebo or drug–drug differences [27,28]. The authors of a recent study with older adults that compared six depression scales concluded that the HAM-D_6_ was the only one to demonstrate total scalability, and that it had the greatest external validity [18].

This scale_,_ may be especially appropriate for use by both older persons and clinicians; its relative brevity makes it comparatively easy for older persons to complete and clinicians to administer. However, to the best of our knowledge, the psychometric properties of responses to the Hebrew HAM-D_6_ had yet to be examined. Thus, the current study examined and compared self-report and clinician responses to the Hebrew HAM-D_6_ for elderly patients.

## Methods

### Scale translation

The HAM-D_6_ was first translated from English to Hebrew by a bilingual psychologist, in keeping with accepted procedures [29]. The translated version was back translated and modified until it was comparable to the original version.

### Training procedures

Two graduate research assistants completed a three-day training course in the administration of study measures. After watching a training tape and receiving instructions, they administered study measures in mock interviews until acceptable inter-rater reliability was established vis-à-vis semi-structured clinical assessments. Research assistants’ HAM-D_6_ scores did not significantly differ from corresponding patient HAM-D responses suggesting no discernible between-rater differences, χ^2^ (*df* = 1) = 1.31, *p* = .25.

### Recruitment

Participants were recruited in the waiting rooms of two primary care clinics operated by Clalit Health Services (Israel’s largest health insurance provided serving 53% of the population). One clinic is located in the center and the other in the north of Israel (Tel Aviv and Haifa, respectively). Inclusion criteria were: 60+ years of age, fluent in Hebrew, and no pronounced cognitive loss (determined using a 6-item screening measure [30]). Participant recruitment took place between May, 2008 and February, 2009.

Research assistants approached patients to request their participation in this study. Participation was voluntary and no remuneration was provided. Those who took part provided written consent. This study was approved by the Helsinki Committee of the Clalit Health Care Services.

### Measures

#### The Structured Clinical Interview for DSM-IV (SCID-I)

The SCID-I is a semi-structured interview to assist clinicians in making a DSM-IV Axis I diagnosis [31]. Only those modules pertaining to depression and dysthymia were administered in the present study. The Hebrew version of the SCID-I was translated and validated by Shalev et al. [32]. All study participants were interviewed using this instrument.

### The 6-item Hamilton (HAM-D6)

The self- and clinician-administered versions of the HAM-D_6_ measure depressed mood, self-esteem and guilt, social interaction and interests, psychomotor retardation, anxiety, and somatic symptoms. Items are provided along 5-point scales, with the exception of the somatic symptoms item (where responses were provided on a 3-point scale). As a screening measure, scores of 7+ suggest clinically significant depressive symptomatology [33]. Whereas the self-report HAM-D_6_ is based solely on patient responses, the clinician-administered version integrates patients’ responses and clinical observation.

### Analytic strategy

We set out to ascertain if the HAM-D_6_ measures a unidimensional construct, as proposed by Bech *et al*. [25]. This hypothesis was tested using confirmatory factor analyses. Both self- and clinician-administered versions of the HAM-D_6_ were next compared to assess the relative contribution of items to measurement (invariance or equivalence analyses). Subsequent analyses were undertaken comparing responses for each patient (self and corresponding clinician HAM-D_6_ responses). Comparisons between SCID diagnoses of a major depressive episode and the patient HAM-D_6_ responses were made to estimate sensitivity and specificity of the scale. Lastly, item-level analyses were computed (intra-class correlation coefficients) to determine if there was agreement between patients and their clinicians for each item.

## Results

This sample was composed of 153 patients 75 years of age on average (range 59–98; SD = 8.1). The majority of participants were male (91/153 or 59.5%). Eighty seven (56.9%) were currently married and living with a spouse, 54 (35.3%) were widowed, and 12 (8.8%) were divorced or lived alone. Respondents’ mean level of education was 11.8 years (range 4–20; SD = 3.1), and the majority (63.4%) ranked their economic status as fair.

### HAM-D_6_ as a screening measure

As previously mentioned, Bech et al. [33] suggest that a HAM-D_6_ score of 7+ is suggestive of clinically significant depressive symptoms (i.e., warranting thorough clinical assessment). Comparing patient and clinician ratings, agreement as calculated using the kappa coefficient was in fair range (k = .26; [34]). Where there was a discrepancy between the two, 13 patients provided responses in clinical range, whereas physicians’ responses indicated these patients were euthymic. A similar finding emerged comparing patient HAM-D_6_ responses with SCID diagnoses of a current major depressive episode (k = .20; linear weighted). Where there was a discrepancy, 14 patients provided HAM-D_6_ responses in clinical range, while the SCID diagnoses indicated no major depressive episode. However, these percentages indicate 100% sensitivity for the patient version of the HAM-D_6_ (true positives) and 91% specificity (true negatives).

### Confirmatory factor analytic models

**Figure 1:**
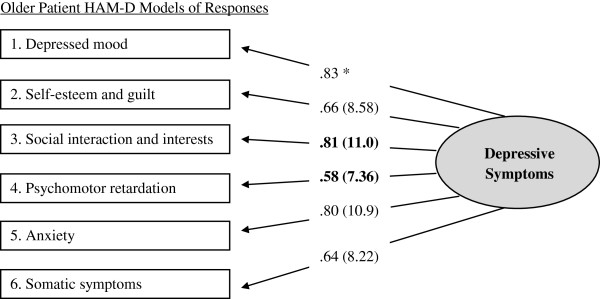
**Older patient HAM-D models of responses. **N*ote*: Maximum likelihood estimates (standardize solution and significance levels). Asterisks (*) denote parameters initially fixed to 1.0 for purposes of scaling and statistical identification. Significance estimates cannot be computed for these two items.

**Figure 2:**
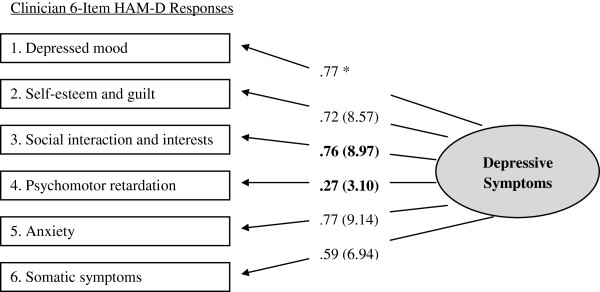
**Clinician 6-Item HAM-D responses. **N*ote*: Maximum likelihood estimates (standardize solution and significance levels). Asterisks (*) denote parameters initially fixed to 1.0 for purposes of scaling and statistical identification. Significance estimates cannot be computed for these two items.

Confirmatory factor analytic (CFA) models were computed separately for older patients (χ^2^*df* = 7] = 23.80, *p* < .01) and corresponding clinician HAM-D_6_ scores, (χ^2^*df* = 9] = 16.93, *p* = .05). Goodness of fit indices for both models were within optimal parameters [35]. Moreover, each of the six items contributed significantly to measurement of a single higher-order construct (i.e., all item *t* values > 1.96); see Figures 1 and 2. For both patient and clinician versions, the HAM-D_6_ appears to measure a unidimensional depression construct.

Next, invariance analyses were undertaken to compare solutions between CFA models. These analyses indicated that responses did not significantly differ for 4 of 6 items. However, responses for the *social interaction and interests* and *psychomotor retardation* items did differ. Both contributed to measurement of depression as reported by patients to a greater degree than that reported by the clinicians. See Table 1.

**Table 1:** Invariance analyses of older patient and clinician 6-Item HAM-D responses

**Model**	**df**	**χ**^**2**^	**Δdf**	**Δ χ**^**2**^	**AGFI**	**SRMR**	**CFI**	**RMSEA**
								**(RMSEA-CL**_**90**_**)**
1. Baseline	17	30.745	–	–	.93	.0352	.98	.052 (.020 – .081)
2. Self-esteem and guilt	18	31.901	1	1.156	.93	.0348	.98	.051 (.019 – .079)
3. Social interaction and interests	19	36.796	1	4.896 **	.92	.0348	.97	.056 (.028 – .083)
4. Psychomotor retardation	20	67.602	1	30.806 **	.86	.0746	.93	.089 (.066 – .113)
5. Anxiety	21	69.090	1	1.488	.87	.0756	.93	.087 (.065 – .110)
6. Somatic symptoms	22	69.127	1	0.037	.87	.0759	.93	.084 (.062 – .107)

*Note*. df = degrees of freedom, AGFI = Adjusted Goodness of Fit Index, SRMR = Standardized Root Mean Residual, CFI = Comparative Fit Index, RMSEA = Root Mean Square Error of Approximation, CL_90_ = 90% Confidence Limits.

### Intra-class correlation coefficients

Intra-class correlation coefficients (ICC) were next computed to directly compare HAM-D_6_ ratings for patient–clinician pairings (i.e., patient self-report vs. corresponding clinician ratings for that patient). ICC values were within adequate parameters for items 1–3 (depressed mood, self-esteem and guilt, social interaction and interests), low for items 5–6 (anxiety, somatic symptoms), but very low for item 4 (psychomotor retardation). This is consistent with invariance analyses reported above, see Table 2.

**Table 2:** **Intra-class correlation coefficients between older patient and clinician HAM-D**_**6 **_**responses**

**Item**	**ICC**
1. Depressed mood	.78
2. Self-esteem and guilt	.73
3. Social interaction and interests	.74
4. Psychomotor retardation	.12
5. Anxiety	.62
6. Somatic symptoms	.64

## Discussion

The goal of this study was to assess the psychometric properties of self-report vs. clinician versions on the Hebrew HAM-D_6_. Results indicated that each of the six scale items contributed significantly to the measurement (both for patients and clinicians) and that HAM-D_6_ responses indeed measure a single depression construct. These findings are in accord with previously reported findings [13-15,25,26,33].

Comparing clinician and patient HAM-D_6_ responses indicate satisfactory correspondence between the two. Moreover, when patient HAM-D_6_ responses were compared to SCID diagnoses of major depressive episodes, sensitivity and specificity were measured as 100% and 91%, respectively.

These findings suggest that a 7+ HAM-D_6_ score is an effective threshold value. Most notably, responses by older adults, themselves, enable effective depression screening between euthymic patients and those reporting pronounced depressive symptomatology.

In addition, findings indicate that responses do not differ significantly for 4 of the 6 items suggesting that patients and clinicians appear to interpret and respond to these HAM-D_6_ items in a consistent manner. Furthermore, the intra-class correlations for 5 of the 6 items were found to be above 0.60. This congruence between patients and clinicians for most scale items implies that patients’ responses can be trusted and accepted as a valid evaluation of depression.

Responses do differ, however, for the *social interaction and interests* and *psychomotor retardation* items. For both items, patients’ responses contributed more to the measurement of depression than clinicians’ responses. Furthermore, the intra-correlation coefficient for the *psychomotor retardation* was found to be very low, but for the *social interaction and interests* item, an adequate correlation emerged.

In light of these intriguing results, we re-examined the Hebrew translations in order to ascertain where refinements are warranted. In English, the second response option for the *social interaction and interests* item reads: “I have felt that I have had difficulty performing my daily activities, but I was still able to perform them with great effort.” The current Hebrew wording translates to: “I had difficulty performing my daily activities, but I was still able to perform routine activities”.

The fourth response of this item in English reads: “I have not been able to do any of the simplest day-to-day activities without help,” and the current Hebrew wording translates to: “I have not been able to do any of the simple day-to-day activities without help.” Although the difference appears minimal, it might have had an effect on the results.

In English, the third and fourth response options for the *psychomotor retardation* item reads: “I have felt clearly slowed down or subdued or have been talking much less than usual,” and “I have hardly been talking at all or feel extremely slowed down at the time.” The corresponding Hebrew wording translates to: “I have felt clearly slowed down or passive and have been talking much less than usual,” and “I have hardly been talking at all and feel extremely slowed down all the time.” We recommend that corrections in translation be made for future studies using the self-report Hebrew HAM-D_6_.

Several limitations of the study need to be acknowledged: a) we do not have data on non-participants and cannot compare this group to our sample, b) we do not have medication data for this sample, c) this is a relatively small sample size, and d) the research assistants that assessed the participants SCID were aware of their HAM-D_6_ scores. Therefore, future studies need to examine the Hebrew HAM-D_6_ with larger samples of participants from different age groups derived by random recruitment.

## Conclusion

Nonetheless, in the light of our results, the Hebrew HAM-D_6_ can be used to measure and screen depressive symptoms among elderly persons. Future psychometric research is required to ascertain whether the above suggested revisions will further improve the psychometric properties of responses to this Hebrew version of the HAM-D_6._

## Competing interests

The authors declare that they have no competing interests.

## Authors’ contribution

YGB wrote the manuscript and made critical revisions. LA, MG and PB conceived, developed and designed the study. LA also supervised the data collection. NO’R carried out the data analysis, wrote the results section and made critical revisions. All authors have read and approved the final manuscript.

## Pre-publication history

The pre-publication history for this paper can be accessed here:

http://www.biomedcentral.com/1471-244X/13/2/prepub
